# The Significance of 8-oxoGsn in Aging-Related Diseases

**DOI:** 10.14336/AD.2019.1021

**Published:** 2020-10-01

**Authors:** Xinmu Zhang, Lin Li

**Affiliations:** Department of Medical Oncology, Beijing Hospital, National Center of Gerontology, Beijing, China

**Keywords:** RNA oxidative damage, 8-oxoGsn, aging-related diseases

## Abstract

Aging is a common risk factor for the occurrence and development of many diseases, such as Parkinson’s disease, Alzheimer’s disease, diabetes, hypertension, atherosclerosis and coronary heart disease, and cancer, among others, and is a key problem threatening the health and life expectancy of the elderly. Oxidative damage is an important mechanism involved in aging. The latest discovery pertaining to oxidative damage is that 8-oxoGsn (8-oxo-7,8-dihydroguanosine), an oxidative damage product of RNA, can represent the level of oxidative stress. The significance of RNA oxidative damage to aging has not been fully explained, but the relationship between the accumulation of 8-oxoGsn, a marker of RNA oxidative damage, and the occurrence of diseases has been confirmed in many aging-related diseases. Studying the aging mechanism, monitoring the aging level of the body and exploring the corresponding countermeasures are of great significance for achieving healthy aging and promoting public health and social development. This article reviews the progress of research on 8-oxoGsn in aging-related diseases.

8-oxo(d)G, namely, 8-oxo-7,8-(2'-deoxy) dihydroguanine /8-oxidized guanine and also known as 8-hydroxy (deoxy) guanine, is a base adduct produced by guanine (G) after exposure to oxidants [1,2]. Because of its quantity and mutagenicity, it is currently the most studied product of nucleic acid oxidation. The 8-oxo(d)G produced by oxidation is no longer paired with cytosine (C) but is paired with adenine (A). Thus, G:C pairing is transformed into A:T pairing via two replication cycles, resulting in gene mutation [3-6].

Harman [7] put forward the free radical theory of aging in 1956, assuming that free radicals may oxidize lipids and proteins and be responsible for aging-related functional degradation. In 1994, another study [8] found that oxidative damage of nucleic acids was found in the brain tissues of Alzheimer’s disease (AD) patients prior to protein and lipid damage. In 1999, Nunomura A *et al.* [9] used an in situ method to identify the oxidized nucleosides 8-oxodG and 8-oxoG in AD patients to determine whether the nucleus and mitochondrial DNA and RNA of AD patients were damaged. It was found that the immune response of patients pretreated with RNase was greatly weakened, but it was only slightly inhibited by DNase. It was concluded that oxidized nucleosides in AD patients were mainly related to RNA. This was the first evidence of increased RNA oxidation in vulnerable neurons in AD and revealed that RNA oxidation is a significant feature of vulnerable neurons in AD. Since then, the oxidative damage caused by nucleic acid has been gradually recognized. At present, more than 20 oxidative bases have been found [10,11]. Among them, guanine has the lowest redox potential and is most vulnerable to oxidative damage. Therefore, 8-oxo(d)G is one of the most prominent lesions in nucleic acid oxidation [12,13]. Because RNA is single-stranded and histone-free and RNA polymerase lacks corrective functions, the oxidative damage of RNA may be more serious than that of DNA [14-18], which has been proven by a large number of studies [19-24]. mRNA damage may lead to abnormal protein translation; tRNA and rRNA damage may lead to dysfunction of protein synthesis [25-29], eventually leading to the production of abnormal proteins, and it may participate in the occurrence and development of diseases. Subsequent studies have proven this hypothesis [9,30,31]. For instance, the accumulation of 8-oxoG in mRNA can lead to the synthesis of pathogenic proteins in mammalian cells [32]. In human and various animal models, the 8-oxo-Gsn (8-oxo-7,8-dihydroguanosine, the final product of 8-oxoG) content in tissues and body fluids is generally higher than the 8-oxo-dGsn content, and the 8-oxoGsn level in urine is the highest [33-38]. The accumulation of 8-oxoGsn is currently recognized as a marker of oxidative stress.

There are two ways to detoxify and inhibit the 8-oxoG mutation in the human body:

1) Nucleotide level: There are two types of 8-oxodG mutant repair during DNA replication: one is the direct removal of 8-oxodG by the DNA glycosylase OGG, and the other is the removal of adenine paired with 8-oxodG by the DNA glycosylase MYH and then the removal of 8-oxodG by OGG to complete the repair process [6,39-43].
2) RNA level: Y-box binding protein-1 and polynucleotide phosphorylase (PNP) specifically recognize and bind 8-oxoG-containing RNA and participate in its degradation, thus maintaining the accuracy of transcription and translation [44,45]. Recent studies have found that two protein factors, namely, AUF1, which can participate in the specific degradation of oxidized RNA, and PCBP1, which can bind oxidized RNA and induce cell death, can remove severely damaged RNA [46].

8-oxoGTP is finally degraded to 8-oxoGsn under the action of MYH, OGG homologous protein MTH1 (NDUDT1), MTH2, NUDT5, and NUDT18. The molecular weight of 8-oxoGsn is 299 g/mol, and it is not further metabolized [47-53]. It is released into the blood as a small molecule and excreted through urine. It has a long half-life in blood and can reflect the oxidative damage of RNA and the oxidative stress level in vivo through tissue, blood, cerebrospinal fluid (CSF) and urine detection [54,55]. Free 8-oxoGsn in urine is considered one of the best biomarkers in the pathophysiological process [12,54]. Quantitative detection of 8-oxoGsn in urine by isotope dilution high-performance liquid chromatography-tandem mass spectrometry (ID-LC-MS/MS) has become the gold standard for the detection of oxidative metabolites of nucleic acids [36-38,57] (Fig. 1).

**Figure 1.**
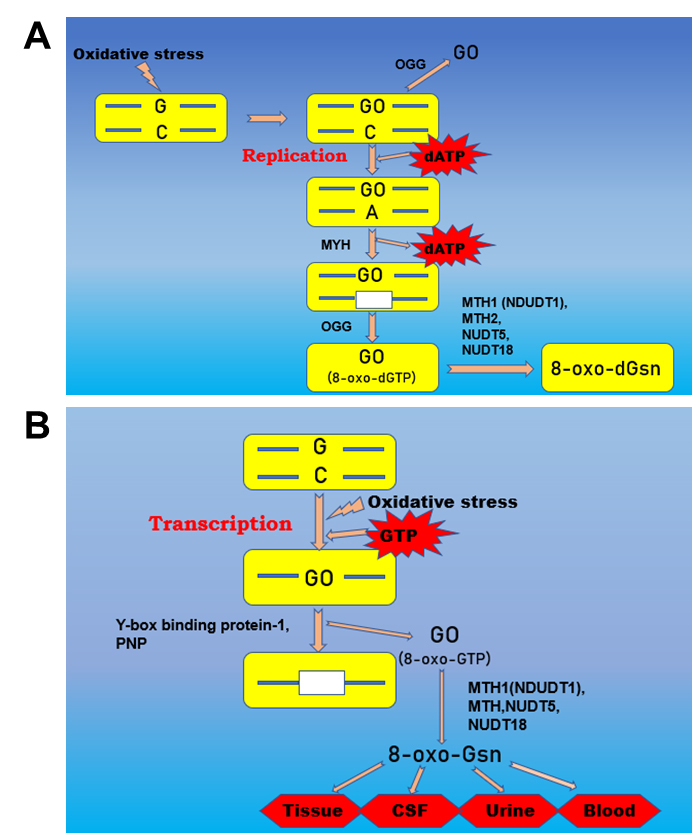
The formation of 8-oxo(d)Gsn. (Up panel) Deoxyguanine (dG) in double-stranded DNA becomes 8-oxodG(GO) under oxidative stress. At this time, 8-oxodG can be directly removed by OGG, or during DNA replication, 8-oxodG is paired with adenine (A) instead of cytosine (C), MYH excision of A is paired with 8-oxodG, and then OGG excision of 8oxodG occurs. (Botton panel) During DNA transcription, G becomes 8-oxoG under oxidative stress, and 8-oxoG is specifically bound and degraded by Y-box binding protein-1 and PNP. The removed 8-oxo(d)G is transformed into 8-oxo(d)Gsn by MTH1, MTH2, NUDT5, and NUDT8 and then enters into tissues, cerebrospinal fluid, blood and urine.

## Progress in the research of 8-oxoGsn in nervous system diseases

RNA oxidative damage has been reported in most common aging-related neurodegenerative diseases, such as Alzheimer’s disease, Parkinson’s disease (PD), Lewy body dementia and amyotrophic lateral sclerosis (ALS) [58-69].

### Alzheimer’s disease and Parkinson’s disease

In 1999, Nunomura A [68] and Zhang J [71] examined nucleotides extracted from brain samples of patients with neurological diseases after death. In the brain tissues of AD and PD patients, the expression level of 8oxodGsn/8-oxoGsn was increased in vulnerable neuron populations, and it was proven that the oxidized nucleosides were mainly 8-oxoG, which is related to RNA [30,72,73]. In 2000, one study [72] found that the level of nucleic acid oxides in cerebrospinal fluid could reflect the degree of nucleic acid oxidation in brain tissue. To further prove the role of RNA oxidation in the pathogenesis of AD and PD, a study [75,76] determined the concentration of 8-oxoGsn in the CSF of PD patients. It was found that the concentration of 8-oxoGsn in the CSF of PD patients was 3 times that of the age-matched controls and was approximately five times as high in patients with AD compared with that of the controls. The concentration of 8-oxoGsn increased significantly in the CSF of patients with AD and PD, suggesting that RNA oxidation may be important in the pathogenesis of AD and PD.

In 2003-2006, to study the role of RNA oxidation in the pathogenesis of AD, one team [77,78] developed and reported a new immunoprecipitation method to isolate oxidatively damaged RNA. It was found that up to 30-70% of the messenger RNA (mRNA) in the frontal cortex of patients with AD was oxidized. The results of the study on oxidatively damaged mRNAs showed that oxidized mRNAs could not be correctly translated, resulting in reduced protein expression and loss of normal protein function. In 2007, the team [79] reported further studies on the relationship between RNA oxidation and neuronal degeneration, revealing that RNA oxidation mainly occurred in a group of neurons that died later, that is to say RNA oxidation was an early event before cell death rather than the result of cell death. The oxidative bases in the RNA cause ribosome transcription arrest, resulting in a significant decrease in the corresponding protein expression level. These studies further confirmed that RNA oxidation is directly involved in neuronal degeneration rather than a harmless side effect in the process of neurodegenerative diseases.

A study [80] published in 2014: to study the levels of nucleic acid oxidation in early and late AD, the team used gas chromatography/mass spectrometry to quantify the levels of multiple base adducts in age-matched normal control subjects (NC), patients with mild cognitive impairment (MCI), non-AD neurological patients (disease control, DC) and patients with preclinical AD (PCAD) or late-stage AD (LAD) in the superior and middle temporal gyrus (SMTG), inferior parietal lobe (IPL) and cerebellum (CER). In subjects with many stages of AD and DC, the median level of base adducts increased significantly (p<0.05); in the SMTG patients, the levels of oxidized nucleic acid base adducts expressed as a % of NC were NC 100.0 [37.4-337.9] *vs.*PCAD 492.8 [118.9-4751.5], MCI 156.1 [78.4-1110.2], LAD 377.5 [101.9-2140.5], and DC 219.0 [62.4-2226.7], suggesting that oxidative damage is not only an early event in the pathogenesis of AD but also prevalent in neuro-degenerative diseases.

### 8-oxoGsn in Atherosclerosis and Coronary Heart Disease

Oxidative stress and intracellular reactive oxygen species (ROS) production have been widely recognized as key factors in the progression of atherosclerosis [81-87]. In the process of studying the relationship between RNA damage and human atherosclerosis, researchers [88,89] found that a strong oxidative damage marker 8-oxoG signal could be detected in smooth muscle cells (SMCs), macrophages and endothelial cells of atherosclerotic plaques, which confirmed the existence of RNA damage in atherosclerotic tissues; however, no evidence of oxidative damage was found in the SMCs of adjacent normal media or mammary arteries. Later, a research team from China [90] found that urinary 8-oxo-Gsn levels and positive rates in patients with coronary heart disease were significantly higher than those in noncoronary heart disease patients. This study suggests that the elevation of oxidative stress in patients with coronary heart disease is of great significance to elucidate the pathogenesis of atherosclerosis and evaluate the condition of coronary heart disease, and a larger sample size is needed to verify these results.

### Study of 8-oxoGsn in Helicobacter pylori (Hp) infection and Hepatitis B virus (HBV) infection in the digestive system

#### Hp infection

Several studies [91-94] reported that Hp infection causes DNA oxidative damage, and the expression of 8-oxodGsn in tissues of patients with Hp infection is significantly higher than that in individuals without Hp infection. Considering that RNA is more vulnerable to oxidative damage and its repair ability is at a disadvantage, a research team [95] from Peking University explored the effect of Hp infection on RNA oxidative damage. They found that 8-oxoGsn, a marker of the local RNA oxidative level, was elevated in patients with Hp infection, compared with normal gastric mucosa, while the immunohistochemical-positive staining OD value of the DNA oxidation level marker 8-oxo-dGsn was not significantly different from that of the normal control group. It is presumed that Hp infection leads to an increase in the level of RNA oxidation in human gastric mucosa. Follow-up studies [96] also found that the level of 8-oxo-Gsn in urine was consistent with that in gastric mucosa. The level of DNA and RNA oxidative damage in urine after Hp infection was higher than that in the Hp-negative control group; in addition, the 8-oxoGsn content was higher. It was speculated that urine detection was more sensitive than histological detection. Urine 8-oxoGsn might be a more sensitive indicator of the pathogenesis of Hp infection.

#### HBV infection

Oxidative damage plays an important role in the occurrence and development of liver injury [97,98]. Previous studies [99] have reported DNA oxidative damage and the accumulation of 8-oxo-dGsn in chronic hepatitis tissues. A clinical observation [100] of chronic hepatitis B patients found that 8-oxoGsn in urine could better reflect the degree of liver inflammation and had better predictive value for the degree of inflammatory activity and fibrosis than pathological results and biochemical indicators, such as Alanine Aminotransferase (ALT) and Aspartate Aminotransferase (AST). Urinary 8-oxoGsn may be an indicator of the severity of liver injury caused by HBV infection and has potential for use in the noninvasive routine clinical detection of liver injury [101].

#### Progress in the research of 8-oxoGsn in Diabetes Mellitus

Over the past decade, the role of oxidative stress in the development of diabetes mellitus has been gradually determined, and oxidative stress has been recognized as the main factor affecting the development of diabetes mellitus [102-105].

Kasper Broedbaek *et al.*[106] conducted research on the oxidative stress level of diabetic patients. First, data from 1381 newly diagnosed type 2 diabetes mellitus patients (excluding exclusive factors) aged over 40 years were retrospectively analyzed. After multivariate correction, the risk ratios of all-cause mortality and diabetes-related mortality in high-quartile 8-oxoGsn patients compared to those in low-quartile 8-oxoGsn patients were 1.44 (1.12-1.85) and 1.54 (1.13-2.10), respectively, and the highest quartile mortality in 8-oxoGsn patients was nearly 50% higher than that in the lowest quartile group. It was confirmed that the urinary RNA oxidative marker 8-oxoGsn was an independent prognostic factor for type 2 diabetes mellitus, while the DNA oxidative marker 8-oxodGsn was not significant. The team further studied [107,108] data from 970 diabetic complications screened during regular follow-up. They found that there was a correlation between the change in 8oxoGsn and mortality from diagnosis to the 6-year follow-up. Increased 8-oxoGsn increased patients' risk of death, while decreased 8-oxoGsn decreased patients' risk. This study revealed that 8-oxoGsn is an exact mechanism for further study of the relationship between oxidative stress and mortality in diabetic patients.

Professor Lanlan Wang of West China Hospital of Sichuan University and Professor Jianping Cai of the National Geriatric Medical Center jointly evaluated the relationship between nucleic acid oxidation and complications in type 2 diabetes mellitus [109]. A total of 1316 subjects were enrolled, including those with type 2 diabetes mellitus and an age- and sex-matched healthy control group. The levels of 8-oxodGsn and 8-oxoGsn in urine were evaluated. Meanwhile, blood glucose, glycated hemoglobin, total cholesterol, high-density lipoprotein cholesterol, low-density lipoprotein cholesterol and triglycerides were determined. The results showed that urinary 8-oxoGsn and 8-oxoGsn levels in diabetic patients with or without complications were significantly higher than those in healthy controls. On the premise of no difference in blood glucose and lipid concentration, the level of 8-oxoGsn in patients with complications, especially those with macrovascular complications, was higher than that in patients without complications. Among them, the increase in 8-oxoGsn was more significant than the increase of 8-oxodGsn. This finding confirms the role of oxidative damage of DNA and RNA; nucleic acid oxidation, especially RNA oxidation, may be one of the molecular mechanisms leading to the progression of type 2 diabetes. Increased levels of 8-oxoGsn may be a risk factor for complications of type 2 diabetes, especially diabetic macrovascular complications.

These studies suggest that the measurement of 8-oxoGsn in urine provides additional information for risk prediction. The combination of 8-oxoGsn and other known risk factors in risk factor analysis may improve risk stratification in diabetic patients. The potential clinical application of 8-oxoGsn as a biomarker in diabetes mellitus needs further study in risk stratification, disease progression, selection of appropriate treatment interventions and monitoring of the treatment response.

#### 8-oxoGsn in Kidney disease

It has been proven that oxidative stress is involved in chronic kidney disease and diabetic nephropathy [105,110-112]. The level of RNA oxidation in patients with kidney disease is higher than that in healthy people. Regarding the retention of urine samples, one study [113] compared the levels of 8-oxo-dGsn and 8-oxoGsn in random urine samples and 24-hour urine samples of healthy subjects and nephrotic patients and found that there was no significant difference between creatinine-corrected levels of nucleic acid oxidation products in random urine samples and 24-hour urine samples, regardless of age group or kidney disease. That is, the levels of 8-oxo-dGsn and 8-oxoGsn in random urine samples can replace the levels of 8-oxo-dGsn and 8-oxoGsn in 24-hour urine samples and can reflect the levels of oxidative stress within 24 hours.

To investigate the relationship between 8-oxoGsn and chronic kidney disease (CKD), one study [114] enrolled 146 patients with CKD from January 2015 to December 2016. Age and sex bias were excluded. In total, 30, 30, 31, 30 and 25 patients with CKD in stages 1-5, respectively, were collected. Fasting blood and morning urine samples were collected and analyzed by ID-LC-MS/MS. It was found that RNA oxidation in CKD patients increased with age and that 8-oxoGsn in the urine of patients older than 60 years increased significantly (P < 0.05). Multiple linear regression analysis showed that 8-oxoGsn was correlated with only serum creatinine (β = 0.656, t = 8.275, P < 0.001). RNA oxidation is closely related to the occurrence and development of kidney diseases. As the disease progresses, RNA oxidation becomes more serious. Significant elevation of 8-oxoGsn in blood/urine is of value for evaluating end-stage renal disease.

#### 8-oxoGsn in Tumors

Tumor gene repair is the premise of maintaining genome integrity and organism health. The accumulation of gene mutation damage is inevitably related to the occurrence of cancer, such as EGFR, K-RAS, ROS-1 and lung cancer; BRCA1/2, PIK3CA hotspot mutations (N345K, E542K, E545K, H1047L and H1047R), PIK3R1 gene mutations (E160D, Q329L, D560Y) and breast cancer; IDH1 R172 mutation and glioma; and somatic mutations of GUCY2F, EPHA3 and NTRK3 mutations and the occurrence of breast cancer, lung cancer and pancreatic cancer [115-117]. Site-specific oxidation of the p53 tumor suppressor gene has been reported at known mutation hotspots, and the codon sites also depend on the type of oxidants [118]. Although the significance of RNA damage has not been fully explained, it can be imagined that RNA damage may lead to abnormal protein translation, while tRNA and rRNA damage may lead to protein synthesis dysfunction, which is also of great significance.

#### Gastric cancer

In 2014, one study [119] observed an increase in the expression of 8-oxo-dGsn in gastric cancer. In 2016, another study[120] performed a comparative analysis of surgical excisions of human gastric cancer and its corresponding adjacent tissues and showed that the level of RNA oxidative stress in cancer cells increased significantly and that 8-oxoGsn accumulated significantly in cancer tissues. This study also observed that the expression of 8-oxoGsn was high in well-differentiated gastric cancer tissues but low in poorly differentiated gastric cancer tissues; that is, 8-oxoGsn is related to the specificity of gastric cancer tissue differentiation. The expression of 8-oxo-dGsn was also higher in gastric cancer tissues than in adjacent tissues. It can be inferred that the occurrence of cancer is related to oxidative stress. Additionally, the growth of cancer tissue is a process of carcinogenesis of surrounding tissue at the metabolic level.

#### Brain tumor

Takashi Iida *et al.* [121] observed the accumulation of 8-oxo-dGsn and the expression of hMTH1 in 42 neuroepithelial tumors, 5 meningiomas, 2 metastatic brain tumors and 1 schwannoma. The accumulation of 8-oxo-dGsn and the expression of hMTH1 in high-grade gliomas were the most obvious results, indicating that the oxidative stress level of gliomas was higher. Therefore, the defense mechanism of antioxidant stress may be enhanced. These results suggest that oxidative stress may play a role in the progression of gliomas.

#### Pan cancer

In terms of tumors, one team from Poland [122] studied 222 cases of head and neck tumors, lung cancer, breast cancer, colon cancer and other tumors and found that urine 8-oxoGsn and 8-oxo-dGsn levels in cancer patients were significantly higher than those in healthy controls and more sensitive than those in peripheral blood. This result indicates that oxidative stress levels in cancer patients increase not only in affected tissues but also in other tissues and the whole body. The problem with this study is that 8-oxoGsn is a marker of aging, thus age is the interference factor in this study; however, this study is not stratify by age. Therefore, a method that can inhibit age-related background levels of oxidative stress markers (8-oxoGsn and 8-oxo-dGsn in urine and 8-oxo-dGsn in leukocyte DNA) may be developed for the early detection of cancer.

#### Discussion

The aging of organisms must be accompanied by the occurrence of oxidative stress and the production of aging damage products. The accumulation of 8oxoGsn in aging-related diseases has been increasingly confirmed clinically. It has been observed that the expression of 8-oxoGsn in disease groups is significantly higher than that in the age-matched control groups in many aging-related diseases and may be related to poor prognosis. Clinicians, especially those who specialize in geriatrics, should be aware of this index when considering adverse prognoses and prognosis in patients. With regard to the study of 8-oxoGsn, the next task should be to further clarify the reference values of the background level of 8-oxoGsn in different age groups, exclude the interference of age-related factors in assessing the severity of disease, and then assess the oxidative stress state of the body and even the endogenous regulation of the oxidative defense system. Developing 8-oxoGsn as an independent disease surveillance index is of great significance for screening aging-related diseases. Urine 8-oxoGsn is a new noninvasive biomarker with excellent sensitivity. It is urgently needed to explore and develop the predictive value of 8-oxoGsn for disease progression and clinical application.

Under the background of the rising incidence of malignant tumors in the world, cancer-related issues have always been a hot topic. It is a challenge and an opportunity to further clarify whether the accumulation of 8-oxoGsn is universally related to the occurrence of cancer, elucidate the relationship between urinary 8-oxoGsn level and the location of tumors, determine whether 8-oxoGsn is the product of pathological changes in tumors or the promoter of tumorigenesis, and determine whether it has the potential to be developed as an independent marker of early cancer screening and a prognostic indicator of tumors. Additional samples and more data are needed for verification, which is the key to future research.

We look forward to the further development and clinical application of markers related to aging.
